# The Efficacy of Wide-Awake Local Anesthesia No Tourniquet (WALANT) in Common Plastic Surgery Operations Performed on the Upper Limbs: A Case–Control Study

**DOI:** 10.3390/life13020442

**Published:** 2023-02-04

**Authors:** Konstantinos Seretis, Anastasia Boptsi, Eleni Boptsi, Efstathios G. Lykoudis

**Affiliations:** Department of Plastic Surgery, Medical School, University of Ioannina, 45100 Ioannina, Greece

**Keywords:** WALANT, upper limb, flap, skin grafting, plastic surgery, local anesthesia

## Abstract

Background: The wide-awake local anesthesia no tourniquet (WALANT) technique is commonly used in elective hand surgery, whereas its application in plastic surgery is still limited. The aim of the study is to evaluate the feasibility and efficacy of WALANT in common plastic surgery operations performed on the upper limbs. Methods: Patients who underwent those operations under WALANT were matched and compared with patients who had general or regional anesthesia without infiltration of a local anesthetic solution. All operations were performed by the same surgeon. Data from 98 operations were collected and analyzed for the total operation time, operation theatre time and complication and patient satisfaction rates. Results: All operations under WALANT, mainly skin tumor excision and flap repair or skin grafting and burn escharectomy with or without skin grafting, were completed successfully. No statistical difference in total operation time and complication rates was revealed. Statistical significance favoring WALANT was identified regarding the mean operation theatre time and patient satisfaction. Conclusions: WALANT is an effective method for common plastic surgery operations performed on the upper limbs that is associated with better operation theatre occupancy and high patient satisfaction rates.

## 1. Introduction

Surgeries in upper limb are usually performed under general or regional anesthesia and include the use of a tourniquet to create a bloodless surgical field [1]. This method, though routinely performed, is associated with certain disadvantages and complications, from pain and damage of anatomic structures to nausea and vomiting postoperatively [2,3].

Wide-awake local anesthesia no tourniquet (WALANT) was proposed by Lalonde in 2005 in order to overcome these limitations [4]. WALANT is based upon the injection of a solution comprising of lidocaine and epinephrine buffered with sodium bicarbonate, providing adequate local anesthesia along with vasoconstriction [5]. The injection of epinephrine in microvascular and hand surgery was initially encountered with fear, due to the potential tissue necrosis caused by epinephrine [6]. However, large studies have demonstrated the safety of epinephrine in finger and hand surgery [4,7]. Phentolamine was proposed as an antidote to epinephrine’s effect on circulation [8].

Even though WALANT was first described by plastic surgeons for certain wrist and hand operations, the technique appealed mostly to hand surgeons, and thus has been principally used in hand procedures, such as trigger finger release, ganglion and cyst excision, fixation of hand fractures, arthroplasties, osteotomy, tendon and ligament repairs, and Dupuytren’s contracture release [1,3]. In addition, carpal tunnel release (CTR) is regularly performed through wide awake anesthesia [9]. McKnight et al. recently reported WALANT outcomes in 1011 elective hand cases, showing safety, low infection rates, cost effectiveness and high patient satisfaction [10]. However, skin grafting and flap harvesting under WALANT, although being a feasible procedure, has had limited application so far [6].

The aim of this study is to present the use of WALANT in common plastic surgery operations performed on the upper limbs and evaluate its feasibility and efficacy in terms of uneventful operation completion, total operation and theatre time and patient discomfort and satisfaction. The hypothesis was that WALANT is a viable option in reconstructing a broad spectrum of skin and soft tissue defects of the upper limbs.

## 2. Materials and Methods

A prospective observational non-randomized comparative study was conducted in the plastic surgery department of a tertiary university hospital between June 2019 and July 2022. The study protocol conformed to the ethical guidelines of the 1975 Declaration of Helsinki, was approved by the local ethical committee and adhered to the STROBE statement for case–control studies. The study was registered in an open access registry, namely the Clinical Trial registry, under the trial number NCT04992351.

Inclusion criteria were adults with American Society of Anesthesiologists (ASA) physical status score 1–3 indication for flap or skin grafting of the upper limb, operated on by the first author. WALANT was used exclusively as the anesthesia method if the patient provided informed consent (intervention group). Exclusion criteria were ASA status >3, the need for other concomitant operations performed in a location other than the upper limbs (except from a skin graft harvesting), known allergy to any of the ingredients of local anesthesia mixture, extreme anxiety due to surgery or refusal to participate. This cohort was compared with a cohort of patients operated on before the introduction of WALANT by the same surgeon under general or regional anesthesia without infiltration of a local anesthetic solution (control group). A 1:1 matching process, in terms of gender and age, was used to limit the effect of these confounding parameters in the outcomes of interest.

The sample size was calculated using G* Power. Taking into consideration that this is the first comparative study in plastic surgery operations, we reviewed the upper limb literature and used the best available evidence from the fixation of distal radial fractures studies, because the complexity and duration of the procedure better reflects the type of operations performed in this study [11,12]. Calculating the effect size from the available data, defining the alpha error and power as 0.05 and 80%, respectively, and assuming equally sized groups, the test yielded a total sample size of 94, and thus, each group should comprise 47 patients in order an effect to be detected. The planned sample size was increased by 5% to 98 patients, to account for the possibility that patients might prematurely withdraw from the study.

The WALANT group of patients were injected before prepping in a typical manner in the subcutaneous plane, always by the first author. The anesthetic solution always consisted of 80 mL normal saline, 20 mL of 2% lidocaine, 10 mL sodium bicarbonate (8.4%) and 1 mL of 1:1000 epinephrine. Enough was injected based on the basic principles of WALANT [5]. The patient’s limb was prepped in standard fashion and the operation was initiated at least 30 min after the infiltration. The patients were followed up at one and four days and two and eight weeks postoperatively.

A prospectively maintained clinical database was used to collect demographics, clinical and surgical parameters of the study population, identify eligible patients for the control group and perform the matching process. The outcomes of interest were the rate of surgery completion under WALANT, the total operation time, operation theatre time, surgical- and anesthesia-related complications and patient satisfaction. The total operation time was calculated as the difference between skin incision and skin closure in minutes. The operation theatre time was calculated as the difference between entry and exit of the patient from the operation theatre in minutes. Patient satisfaction was estimated by a 10-point visual analogue scale at the eight-week follow-up appointment.

Continuous variables were compared with t test or Mann–Whitney U test according to their distribution, whereas Fisher’s exact test was used for categorical variables using SPSS (Version 27, IBM SPSS Statistics, Chicago, IL, USA). Data were presented as absolute number, percentage, mean and standard deviation (SD). Statistical significance was defined as less than 0.05.

## 3. Results

**Figure 1:**
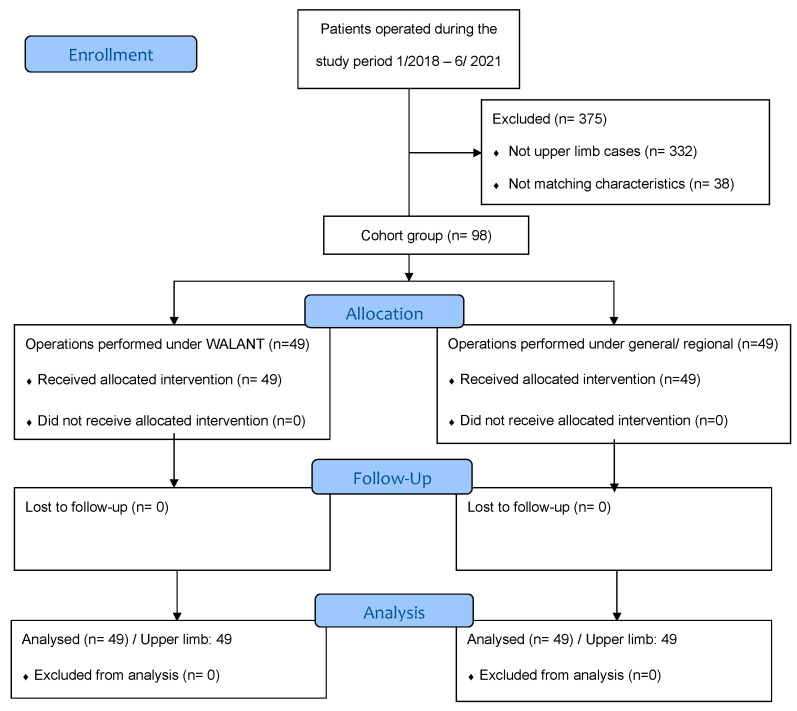
Study flow diagram.

During the study period, 98 operations performed in the upper limb either under WALANT or general/regional anesthesia were included in the study (Figure 1).

### 3.1. Patient Demographics

The patients’ demographic data are shown in Table 1. There were no significant differences between groups in terms of age, gender or upper limb part involvement.

### 3.2. Clinical and Surgical Outcomes of Interest

**Figure 2:**
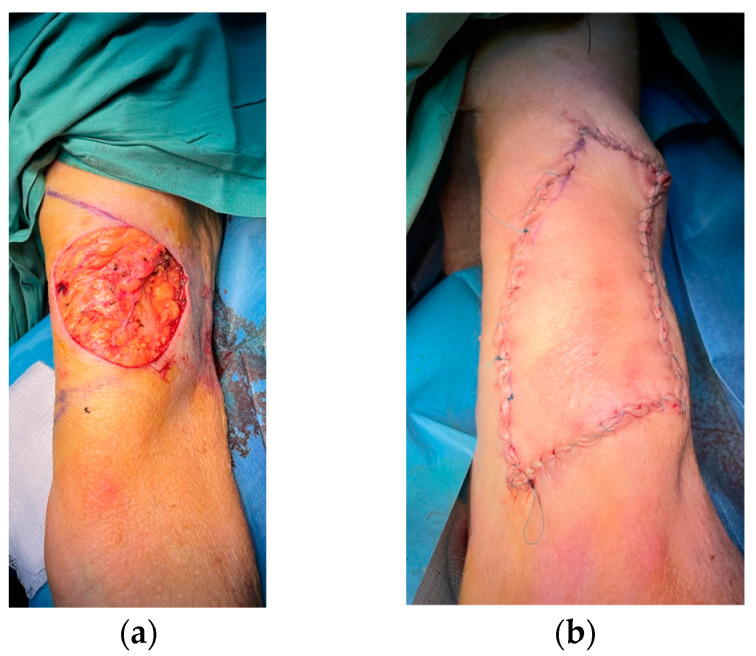
A case of skin cancer excision and reconstruction of the arm defect with a keystone perforator flap: (**a**) Arm defect; (**b**) Immediate postoperative result.

**Figure 3:**
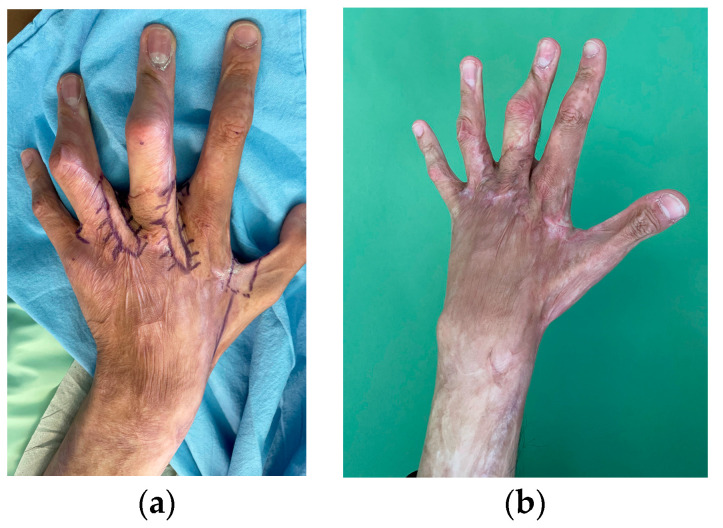
A case of multiple post-burn contractures of the left hand interdigital folds reconstructed with local flaps: (**a**) Preoperative view; (**b**) 3-month postoperative result.

**Figure 4:**
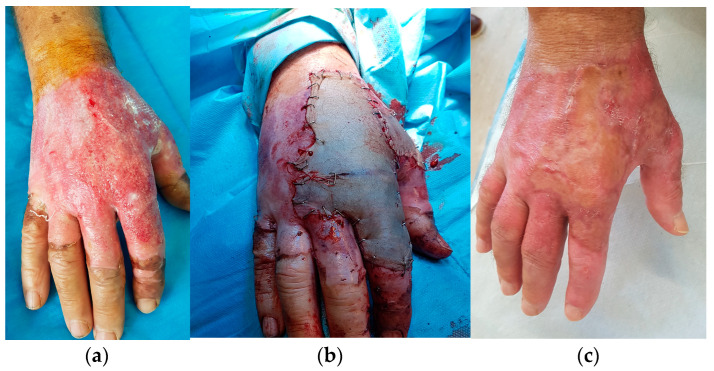
A case of a deep partial thickness burn of the right hand reconstructed with skin grafts: (**a**) Preoperative view; (**b**) intraoperative and (**c**) 2-month postoperative result.

The type of operations performed were mainly skin tumor excision and flap harvesting and insetting or skin grafting, burn escharectomy with or without skin grafting, and scar correction. Local, regional, keystone and perforator flaps were performed under WALANT in 25 cases, and partial- or full-thickness skin grafting was performed in 22 cases (Figure 2, Figure 3 and Figure 4). More than one upper limb operation was performed for three patients in the WALANT (Figure 3) and four patients in the control group.

One case of subcutaneous tumor excision of the forearm with a 10 cm diameter was performed in each group. No significant statistical difference between the two groups in terms of operation type was identified (Table 2). All WALANT cases were completed successfully without emergency conversion to another anesthesia technique, and no intraoperative complications in either group were reported. Four patients (8.2%) required reinjection intraoperatively, due to insufficient anesthesia towards the end of the operation.

The mean total operation time was 56 min (range = 22–89; SD = 18) in the WALANT group and 58 min (range: 26–84, SD = 12) in the control group, which was not statistically significant (*p* > 0.05) (Table 2). The mean operation theatre time was 61 min (range: 24–98; SD = 19) in the WALANT group and 79 min (range: 43–102; SD = 14) in the control group, which was statistically significant.

Three patients reported postoperative nausea and vomiting (PONV) in the control group. Minor surgical complications were recorded in the WALANT (two infections, one dehiscence) and control groups (three infections, one hematoma, one dehiscence) postoperatively, whereas no major complications were recorded. The statistical analysis between the two groups, regarding complications, was not significant.

Patient satisfaction was measured as very high in both groups but favoring the WALANT group (mean score 9.3 ± 1.0 vs. 8.9 ± 0.8, *p* = 0.03).

## 4. Discussion

Modern plastic surgery is characterized by innovation and ingenuity and low complication and high satisfaction rates. These traits are also desirable and thus to be sought in the population of patients who undergo reconstructive operations. In this direction, WALANT has emerged as an attractive alternative technique in upper limb surgery to the traditional application of tourniquet under general or regional anesthesia. Despite its merits, the tourniquet is associated with potential complications, including the development of compartment syndrome, tissue necrosis, nerve damage, postoperative pain and swelling [13,14]. Horlocker et al., reported a strong correlation between nerve injury and prolonged tourniquet time, showing that the risk of neurologic complications is approximately tripled for each 30 min extension of an applied tourniquet [15]. In addition, the general or regional anesthesia used to surpass the pain caused by the tourniquet or the inconvenience from the extended operation field, are associated with their own complication risks. WALANT has been promoted by Lalonde as a safe alternative method for certain hand operations, such as tendon repair and trigger finger and carpal tunnel release among others, providing sufficient anesthesia and vasoconstriction and thus avoiding the discomfort and pain caused by the use of a tourniquet [13]. In addition, remaining awake during surgery, the patient can cooperate by performing active movements, so that the surgeon can better evaluate the surgical outcomes and thus further adjustments and refinements can be employed if required [6,16]. WALANT also presents a safe option for patients with cardiopulmonary comorbidities, for whom general or spinal anesthesia is contraindicated [1]. Taking into consideration these favorable attributes of WALANT, the objective of this study was to explore, objectively, if it can be also employed during skin and soft tissue reconstruction.

This technique would be beneficial, especially for plastic surgeons who are already familiar with the usage of lidocaine–epinephrine solution as a lot of procedures, from cosmetic facial surgery to liposuction and body contouring, are routinely performed following infiltration with local anesthetic [17,18,19]. Tumescent liposuction was described in the mid 1980s and popularized by Klein [20]. At our institution, the composition of the anesthetic solution used is based on the principles established by Klein and Lalonde and adapted according to each patient’s comorbidities and the specific surgical needs. The local anesthetic solution comprises lidocaine and epinephrine mixed with normal saline. Epinephrine, even in low concentrations, provides significant hemostasis, enhances the anesthetic effect of lidocaine as it augments its infiltration in the tissues, and prolongs its duration [19,21]. Moreover, the combination of lidocaine with epinephrine has an anti-inflammatory effect [22]. The solution is buffered with sodium bicarbonate in a ratio of 1:10 to neutralize the acidic pH of lidocaine and eliminate the burning sensation and discomfort during the administration of the solution, whereas a higher pH also accelerates the onset of the anesthesia effect [9,21]. The patient normally feels 1–2 pricks (1.70 on average in the study of Luciani et al.) with a 30-gauge needle, compared with the plexus blocks, which are generally performed with a large-gauge needle, using a triple stimulation technique to optimize results [23,24].

This is the first comparative study evaluating the use of the WALANT approach to excise mostly sizable skin cancers in the upper limbs and subsequently reconstruct the skin and soft tissue defects by means of various flap transfer or skin grafting but also to perform burn escharectomy and skin grafting. Local, regional, keystone and perforator flaps have been successfully used in this study. Partial- or full-thickness skin grafting following skin tumor extirpation or escharectomy has also been successfully performed, avoiding the use of other anesthesia modalities. Compared with the control group, WALANT patients underwent operations in a similar mean operation time, with significantly less total time in operating theater. These outcomes seem contradictory to the relevant results reported by a systematic review and meta-analysis on the outcomes of atraumatic hand surgeries performed using WALANT versus anesthesia and tourniquet, which showed that the WALANT technique was associated with statistically significantly longer operative times. This can be explained by the fact that the injection of the anesthesia was performed directly in the operating theater and as a result, the surgeon had to wait 26–30 min for the adrenaline to cause the necessary vasoconstriction [13]. Unlike this, we performed WALANT in our patients while they were still in the wards and thus reduced the total time in the operation room. Moreover, further time gain was achieved by injecting one case ahead, and then operating sequentially. Lalonde proposes to inject even two cases ahead, should the duration of surgery be short, such as in trigger finger release or CTR, but this approach was not usually feasible in our study, as we had to reconstruct skin and soft tissue defects by means of flaps or skin grafting. The time from infiltration to incision was always more than 30 min, complying with the ideal interval of 26 min reported in the literature and thus ensuring an effective occupation of the operation theatre [25]. This fact is also corroborated by the low intraoperative reinjection rate reported, which was limited to the later stages of an operation.

The complication rate was low, as expected, and similar between the groups, showing the safety of WALANT in this comparative study. No skin or flap necrosis was reported in the WALANT group, further strengthening the concept of safety of the technique in flap harvesting and defect reconstruction. Interestingly, significantly higher satisfaction rates were reported in the WALANT group. Patients preferred WALANT over general or regional anesthesia due to the avoidance of nausea and vomiting, anesthesia-related dizziness postoperatively and the elimination of tourniquet and deep nerve pain [3,9]. In a case–control study, Nikkhah et al., reported a 93% satisfaction rate in patients with carpal tunnel release under WALANT [26]. Furthermore, it offers a better intraoperative experience as the patient can communicate with the surgeon during the procedure and perform active movements when needed [3]. This interaction cements the surgeon–patient relationship and enables intraoperative patient counselling; thus, the process of postoperative instructions becomes individualized, more efficient and less time-consuming for both the surgeon and the patient.

Reviewing the pertinent literature, before this study design and performance, we found that WALANT has not been applied widely in plastic surgery. Tang et al., reported their experience from two large hand-surgery centers in China, where WALANT was applied to more than 12,000 patients over a period of eight years [27]. Initially, the technique was used in the case of emergency hand surgeries, so that the preoperative time was diminished. In fact, the total waiting time from injection to incision was only 5 to 15 min, in contrast with the 26 min delay previously indicated [25]. Similarly, Luciani et al. used WALANT for emergency hand surgery in 58 patients, with 57% of them undergoing simple wound care, 29% tendon repair and the remaining nerve, artery, or bone (osteosynthesis) repair. The mean waiting time was 35.6 min, the mean operating time was 24.6 min and the mean pain score was 0.36 (out of 10), with the pain described as the same or less than that experienced at the dentist by 86.2% of patients [23]. Xing and Tang extended WALANT applications in 27 finger repairs by means of four different flap methods, harvesting and transferring either an extended Segmuller flap, a homo-digital reverse digital artery flap, a dorsal metacarpal artery perforator flap or an Atasoy advancement flap [28]. The authors found that the vasoconstriction caused by epinephrine mainly affects the capillaries, it does not affect the digital arteries and their major branches in the hand, and thus, harvesting a flap in the hand and fingers may not be a contraindication to wide-awake surgery. They also reported that only the dorsal metacarpal artery perforator flap suffered from ischemia, which was later resolved without further surgery by injecting phentolamine intraoperatively. Based on their results, the injection of phentolamine immediately after flap transfer was proposed as a routine approach to most microvascular surgical procedures under WALANT, including digital-artery-based pedicled flaps and perforator flaps in the hand. We can also confirm the feasibility of those flaps’ harvesting and transfer in our cohort without the use of phentolamine. We have also applied WALANT to harvest rather large keystone flaps, type I and IIb, and then reconstruct successfully sizable defects of the arm and lower third of the forearms. Prasetyono et al. suggested that the infiltration of tumescent solution in flap surgery should be performed cautiously [14]. They claimed that the necrosis is caused by the injury of the perforator vessel from the multiple needle insertions rather than the vasoconstrictive effect of epinephrine. We observed neither a flap necrosis nor an increased complication rate compared with the control group. Tang et al. applied local anesthesia with epinephrine in order to perform flap harvest and transfer to cover hand defects in 50 cases and skin grafting in 149 cases, respectively, and reported no vascular complications by the vasoconstriction that epinephrine induced [27]. Similarly, Xu et al. supported the reliability and safety of WALANT in 12 cases with traumatic finger skin defects repaired with random pattern abdominal or thoracic skin flaps [6]. We can also confirm the feasibility of skin graft harvesting under WALANT, usually from a donor area outside the upper limb, such as the thigh or inguinal area, and the successful transfer to the upper limb defect, without any compromise of the functional and aesthetic result.

Studies have also indicated WALANT to be a cost-effective procedure, in terms of preoperative consultations, postoperative recovery and total medical and productivity costs, since there is no requirement for tourniquet and regional or general anesthesia in the main operating room, thus less equipment is needed [3]. It is also worth mentioning that the WALANT approach during the Coronavirus pandemic was recommended by the British Association of Plastic Reconstructive and Aesthetic Surgeons and the British Society for Surgery of the Hand for hand injury management, so that medical resources are conserved [29]. Expecting a surge of surgical cases following the tremendous impact of the early pandemic phase on skin cancer, WALANT seems a viable, reliable and worthwhile procedure [30,31]. Although we have not performed a formal cost-analysis, we estimate lower total costs in the WALANT group due to the significantly shorter operation theater occupation time and the avoidance of the main operating room, the anesthesiologist and most of the non-medical personnel. Leblanc et al. concluded that the performance of a CTR in an ambulatory setting under WALANT is four times less expensive than a CTR in a main operating room [32]. Similarly, Maliha et al. reported that trigger finger release under WALANT decreased the cost to more than 33%, which is in accordance with the cost analysis study of carpal tunnel and trigger finger release published recently [3,33]. Kamnerdnakta et al. performed a retrospective claims analysis of 352,779 patients undergoing minor hand surgery (de Quervain’s tendonitis and carpal tunnel release) and estimated that, over 5 years, a saving of $133 million could have been made by the absence of an anesthetist should these procedures had been performed under WALANT [34]. Although similar outcomes are anticipated by performing very common plastic surgery procedures on the upper limbs, such as skin tumor excision, burn escharectomy and flap reconstruction or grafting under WALANT, a future well-designed cost-effectiveness study could elucidate the economic impact of this procedure.

Despite the advantages of the use of epinephrine in the surgical field, it may be contraindicated for patients who have a history of vascular insufficiency, unilateral or bilateral digital bundle injuries, vaso-occlusive disease, sickle cell disease or other blood dyscrasias and allergies [2]. An alternative option for those patients is a variation of WALANT, described as wide-awake local anesthesia without epinephrine (WALANE) [14]. This technique is based upon the hemostasis caused by the self-retaining retractor and the anesthetic volume injected, which elevates the pressure subcutaneously. Even though this variant was associated with a longer operative time, it ensures sufficient visualization of the surgical field and thus it is considered a feasible alternative option instead of WALANT. Another limitation of epinephrine use in flap harvesting is the difficulty of intraoperatively assessing the flap perfusion either with the capillary refill or the skin color due to its vasoconstriction effect. However, this is surpassed by applying an adequate dosage of the described solution and a meticulous technique, as skin and flap perfusion recovers shortly after the surgery. Xing et al., proposed the routine injection of phentolamine directly after the flap transfer to limit the possibility of flap ischemia or necrosis, which we have never applied, without this approach to increase our complication rates [28]. Furthermore, Zargaran et al. suggested the formation of a WALANT checklist that will include the contraindications of the procedure as well as the recommended dose and indications of phentolamine [35]. Tranexamic acid (TXA) use emerges as a viable option instead of epinephrine, accumulating the evidence from the field of aesthetic plastic surgery in terms of feasibility, safety and complication rates [36]. Consequently, TXA can be alternatively added to the solution performing WALANT in the case of epinephrine contraindication.

The study outcomes are subject to certain limitations, mainly due to the non-randomized control trial design employed. However, it is generally difficult to randomly allocate patients performing most surgical techniques. Aiming to overcome this inherited study limitation, we created homogenous groups, using the process of group matching, with no statistically significant differences concerning the patient and surgery characteristics and included consecutive cases performed by the same surgeon, with the exact same surgical technique, differing only on the anesthesia technique applied. In this way, potential confounding variables, which can increase the study bias, were mitigated, increasing the reliability of the study. In addition, the outcomes of the study could be generalized only to the certain types of operations performed, as defect size and technique complexity could limit its applicability. Patient-related factors, such as known allergy to any of the ingredients of WALANT mixture or extreme anxiety, may also preclude its application. Overall, based on this study and our accumulated experience, using this technique, a broad spectrum of skin and soft tissue upper limb defects can be reconstructed conveniently under WALANT.

## 5. Conclusions

This is the first comparative study of WALANT technique applied in common plastic surgery operations of the upper limbs, including skin tumor excision and concomitant flap repair or skin grafting, burn escharectomy with or without skin grafting, as well as scar correction. It was demonstrated that WALANT provides a safe and well-tolerated approach in such operations, it is time efficient and thus cost-effective compared with regional or general anesthesia, meanwhile offering higher patient satisfaction rates. Therefore, it is proposed as a valuable and attractive alternative option to the tourniquet application in several basic and advanced upper limb plastic surgery operations and a great skill for the plastic surgeon to master. Based on the accumulated experience and the outcomes of this study, WALANT has been implemented as part of our routine practice for common plastic surgery operations performed on the upper limbs. Further studies of high methodological quality are eagerly anticipated to consolidate the advantages of the technique and thus establish its broad application in upper limb skin and soft tissue reconstruction.

## Figures and Tables

**Table 1:** Patient characteristics.

Variables	WALANT	CONTROL	*p* Value
	(N = 49)	(N = 49)	
Age (y)	60.73 ± 18.08	61.04 ± 17.09	0.89
Gender Male Female	28 21	26 23	0.84
Anatomical Region Hand Forearm Arm	34 8 7	27 12 10	0.34

**Table 2:** Perioperative and postoperative data.

Variables	WALANT	CONTROL	*p* Value
	(N = 49)	(N = 49)	
Operation type Flap Skin grafting Escharectomy Other	25 22 1 1	20 25 3 1	0.63
Operation time (min)	56.20 ± 18.15	58.39 ± 12.34	0.49
Operation theatre time (min)	60.63 ± 18.92	78.82 ± 13.90	0.000
Complications No Yes	46 3	44 5	0.72
Satisfaction	9.27 ± 1.02	8.86 ± 0.79	0.03

min: Minutes. *p* < 0.05

## Data Availability

Available by the authors.
